# *Escherichia coli* Producing CMY-2 β-Lactamase in Retail Chicken, Pittsburgh, Pennsylvania, USA

**DOI:** 10.3201/eid1803.111434

**Published:** 2012-03

**Authors:** Yoon Soo Park, Jennifer M. Adams-Haduch, Jesabel I. Rivera, Scott R. Curry, Lee H. Harrison, Yohei Doi

**Affiliations:** University of Pittsburgh School of Medicine, Pittsburgh, Pennsylvania, USA (Y.S. Park, J.M. Adams-Haduch, J.I. Rivera, S.R. Curry, L.H. Harrison, Y. Doi);; University of Pittsburgh Graduate School of Public Health and School of Medicine, Pittsburgh (L.H. Harrison);; Gachon University Gil Hospital, Incheon, South Korea (Y.S. Park)

**Keywords:** cephalosporin resistance, retail chicken meat, CMY-2 β-lactamase, Escherichia coli, bacteria, antimicrobial resistance, United States, Pennsylvania, chicken

**To the Editor:** Rates of resistance to various antimicrobial drugs are rapidly increasing in *Escherichia coli*, not only in health care settings but also in the community. The food supply is suspected as a potential source of antimicrobial-resistant *E. coli* strains, which include cephalosporin-resistant *E. coli* found in retail meat products and other types of food (*1*).

We reported a high prevalence of cephalosporin-resistant *E. coli*, most of which produced CMY-2 β-lactamase, among retail poultry products in Pittsburgh, Pennsylvania, USA during 2006–2007 (*2*). CMY-2 is the most commonly acquired ampicillin C (AmpC)–type β-lactamase found in *E. coli* that cause human infections (*3*). The aim of this study was to investigate whether cephalosporin-resistant *E. coli* are present in retail raw meat and ready-to-eat meat products in our area 5 years after our previous study and define subtypes of concern.

A convenience sampling of 104 raw ground meat products from 3 local grocery stores in Pittsburgh was performed during February–April 2011. Items purchased were samples of all available deli counter ground meat, all fresh sausages prepared in stores at the deli counter, and selected uncooked commercially packaged fresh and frozen sausages. Types of meat items were chicken (n = 22), turkey (n = 10), lamb (n = 2), pork (n = 43), and beef (n = 27).

Approximately 10 g of each sample was excised and suspended in 10 mL of nutrient broth. After being incubated overnight at 37°C, 10 µL of broth was plated on MacConkey agar plates containing 2 mg/L of cefotaxime or ceftazidime, and the plates were incubated overnight at 37°C. Lactose-fermenting colonies were identified as *E. coli* by using standard biochemical methods, which included sulfide indole motility, growth on triple sugar iron medium, oxidase activity testing, and the API20E system (bioMérieux, Durham, NC, USA) as needed.

Antimicrobial drug susceptibility was determined by using the disk diffusion method (AB Biodisk, Solna, Sweden) according to the manufacturer’s instructions and interpreted according to the criteria of the Clinical and Laboratory Standards Institute (*4*). Isolates were screened for extended-spectrum β-lactamase (ESBL) production by using the double-disk diffusion method and for acquired *ampC*-type β-lactamase genes by using multiplex PCR (*4**,**5*).

Phylogenetic groups (A, B1, B2, and D) were determined as reported (*6*). Screening for sequence type (ST) 131 was conducted by using PCR and confirmed by using multilocus sequence typing (*7**,**8*). Pulsed-field gel electrophoresis was performed to determine clonal relationships by using *Xba*I and the protocol available through the PulseNet (www.cdc.gov/pulsenet/protocols.htm). Banding patterns were analyzed by using BioNumerics software version 6.01 (Applied Maths, Sint-Martens-Latem, Belgium).

Among 104 meat samples, 9 contained cephalosporin-resistant *E. coli*, resulting in an overall prevalence of 8.7% (95% CI 4.0%–15.8%). Cephalosporin-resistant *E. coli* was isolated from 7 (31.8%) of 22 chicken, 1 (10.0%) of 10 turkey, and 1 (2.3%) of 42 pork samples. No cephalosporin-resistant *E. coli* was detected from beef and lamb samples. Incidence of samples with cephalosporin-resistant *E. coli* was lower than in our previous study (*2*). This finding may be caused by different types of samples included in the studies or a true decrease in incidence.

Features of cephalosporin-resistant *E. coli* identified are summarized in the Table. None produced ESBL, but all 9 isolates were positive for the CMY-2 β-lactamase gene and positive results were confirmed by sequencing. CMY-2 is the most commonly observed acquired AmpC β-lactamase in *E. coli* and nontyphoidal *Salmonella* species in meat products (*9*).

**Table:** Characteristics of 9 cephalosporin-resistant *Escherichia coli* isolates in retail meat, Pittsburgh, Pennsylvania, USA*

Sample no.	Origin	Phylogenetic group	Susceptibility					
			CTX	FOX	FEP	CIP	GEN	TET
FD13	Chicken (ground)	D	R	I	S	S	S	S
FD14	Pork (sausage)	A	R	R	S	S	S	R
FD42	Chicken (sausage)	B2	R	R	S	S	S	S
FD44	Chicken (thigh)	A	R	R	S	S	S	S
FD45	Turkey (sausage)	A	R	R	S	S	R	R
FD56	Chicken (sausage)	D	R	R	S	S	S	S
FD63	Chicken (sausage)	A	R	R	S	S	S	S
FD72	Chicken (sausage)	A	R	R	S	S	S	R
FD95	Chicken (liver)	A	R	I	S	S	S	S

*All isolates were CMY-2 type. CTX, cefotaxime; FOX, cefoxitin; FEP, cefepime; CIP, ciprofloxacin; GEN, gentamicin; TET, tetracycline; R, resistant; I, intermediate; S, susceptible.

As for the phylogenetic groups, 6 (66.7%) of 9 cephalosporin-resistant *E. coli* belonged to group A, which is generally considered to be a commensal phylogenetic group. However, 1 group B2 isolate from a chicken sample was identified as ST131. The CMY-2 gene was located on an IncI1-type plasmid and was transferable to another *E. coli* strain by conjugation for this isolate. Two group D isolates from chicken that belonged to ST117, which has been reported in ESBL-producing isolates of human and animal origins (*10*); no clonality was observed for the other 7 isolates by pulsed-field gel electrophoresis,

*E. coli* ST131 has emerged as a worldwide pathogen and causes mainly community-onset extraintestinal infections. Although the pandemic spread of *E. coli* ST131 was first identified in isolates producing CTX-M-15 ESBL, it is increasingly recognized that isolates belonging to this clone may also harbor other drug resistance determinants. Among acquired AmpC β-lactamases, CMY-2 has been most frequently reported in ST131 from human clinical isolates (*3*). Infections caused by CMY-producing *E. coli* are common but underrecognized because of the lack of standardized detection methods (*2*).

Given the rapid global spread of the ST131 clone and the possibility of its transmission from food animals to humans, coupled with an abundance of CMY-2–encoding plasmids in poultry environments, *E. coli* ST131 producing CMY-2 β-lactamase may have potential to spread to humans. Our results also show that *E. coli* producing CMY-2 continues to be found commonly among retail chicken products in our study area.
